# Recurrent Cylindrical Battery Ingestion in Borderline Personality Disorder: Balancing Medical Risk and Behavioural Reinforcement

**DOI:** 10.7759/cureus.116845

**Published:** 2026-09-24

**Authors:** Carolina Monteiro, José Castro, Rita Macas, Ana Sofia Barbosa

**Affiliations:** 1 Psychiatry, Hospital Prof. Dr. Fernando Fonseca, Amadora, PRT

**Keywords:** autistic traits, behavioural reinforcement, borderline personality disorder (bpd), cylindrical battery ingestion, deliberate foreign body ingestion, factitious disorder imposed on self, liaison psychiatry, self-harm

## Abstract

Intentional foreign body ingestion is a recurrent and clinically challenging form of self-harm in patients with borderline personality disorder (BPD). Although medical guidance informs the management of individual ingestion events, recurrent presentations raise additional questions about the function of the behaviour and the consequences of repeated medical intervention. We report an 18-year-old patient with BPD and previously documented factitious features who had six episodes of intentional foreign body ingestion during a two-month psychiatric admission in 2026: five involving cylindrical batteries, including re-ingestion of a previously passed battery, and one involving a metal screw. Serial psychiatric assessments identified no psychotic symptoms. The behavioural context evolved from impulsive and reactive self-harm to later episodes that the patient described as retaliatory in the context of perceived interpersonal rejection. Multidisciplinary management integrated established medical indications with longitudinal psychiatric formulation. Endoscopic retrieval was undertaken when clinically indicated, whereas distally progressing batteries were managed conservatively with close clinical and radiological monitoring. No major medical complications occurred. This case illustrates a recurring dilemma at the interface between psychiatric and medical care: medically necessary procedures should never be withheld because of concerns about behavioural reinforcement, but unnecessary intervention may become incorporated into maladaptive behavioural and interpersonal patterns when a safe conservative alternative exists. Psychiatric formulation can therefore complement medical risk assessment and support consistent, proportionate multidisciplinary care.

## Introduction

Intentional foreign body ingestion is a recurrent and clinically challenging form of self-harm, particularly among patients with psychiatric disorders and borderline personality disorder (BPD) [1,2]. Recurrent episodes may result in repeated emergency assessments, imaging, endoscopic procedures, and hospital admissions, with substantial healthcare utilisation. In one retrospective series, 33 patients accounted for 305 intentional foreign body ingestion episodes over approximately eight years, generating estimated healthcare costs exceeding US$2 million [3].

Battery ingestion presents specific management challenges because the risk of injury depends on battery type, anatomical location, integrity, and duration of retention. Button batteries lodged in the oesophagus are particularly hazardous because electrical current can generate hydroxide ions at the negative pole, producing rapidly progressive alkaline tissue injury. Cylindrical batteries generally have a different risk profile and are more likely to progress through the gastrointestinal tract; however, they may still cause significant mucosal injury, particularly when retained for prolonged periods or when the casing is damaged [4,5]. Current endoscopic guidance therefore bases intervention on factors including foreign-body characteristics, anatomical location, symptoms, and progression through the gastrointestinal tract [4].

Although established medical guidance informs the management of individual ingestion events, recurrent intentional ingestion raises additional challenges that cannot be addressed solely through object-based algorithms. In patients with BPD, repeated ingestion may occur within broader patterns of impulsivity, recurrent self-injury, affective dysregulation, and interpersonal difficulties [1,2,6]. Repeated medical intervention may also become incorporated into the behavioural and interpersonal context in which self-harm occurs, creating a need to integrate medical risk assessment with longitudinal psychiatric formulation.

This case report describes recurrent intentional cylindrical battery ingestion during a two-month psychiatric admission in a patient with BPD. It aims to illustrate the clinical and behavioural challenges associated with repeated ingestion and to examine how psychiatric formulation may complement established medical indications in supporting consistent and proportionate multidisciplinary management.

## Case presentation

An 18-year-old transgender male (assigned female at birth) with a diagnosis of borderline personality disorder (BPD) according to the Diagnostic and Statistical Manual of Mental Disorders, Fifth Edition, Text Revision (DSM-5-TR) criteria [7] and previously documented factitious disorder was admitted to an acute psychiatric inpatient unit for approximately two months in 2026 following deliberate multidrug ingestion in the context of suicidal ideation. The patient had been transferred from a residential psychiatric facility. Clinical information for this report was obtained from the patient's medical records, including contemporaneous psychiatric and multidisciplinary clinical notes, endoscopy reports, and serial abdominal imaging. His medical history included Wilms tumour treated surgically during childhood and precocious puberty. His psychiatric history was notable for multiple previous psychiatric admissions for suicidal ideation and severe self-injurious behaviours, including previous intentional foreign body ingestion. He also reported sexual abuse by a family member at approximately seven to eight years of age, with self-injurious behaviours beginning at approximately 10 years of age. At admission, his psychopharmacological regimen included quetiapine, trazodone, topiramate, low-dose venlafaxine, hydroxyzine, and flurazepam. He had also received a six-monthly long-acting paliperidone injection prior to admission.

At admission, the patient presented with persistent suicidal ideation in the context of recurrent self-harm and recent repeated suicidal behaviour. Mental state examination showed depressed mood and a sad affect, with partially preserved insight. No abnormalities of thought form, content or perception suggestive of a psychotic process were identified. During the admission, serial psychiatric assessments similarly identified no psychotic symptoms.

During the approximately two-month admission, the patient engaged in six episodes of intentional foreign body ingestion: five involving cylindrical batteries and one involving a metal screw. The cylindrical batteries were obtained from television remote controls available in patients' rooms on the psychiatric ward, with one remote control present in each room. The metal screw was removed by the patient with his fingers from a window in one of the patient rooms. The chronology, behavioural context, management, and outcomes of the individual episodes are summarised in Table 1.

**Table 1:** Summary of intentional foreign body ingestion episodes during hospitalisation

Time from admission	Foreign body	Behavioural context	Location/course	Management	Outcome
Day 9	2 × AA batteries	Impulsive self-harm; denied primary suicidal intent	Stomach → distal GI tract	Upper gastrointestinal endoscopy; subsequent conservative management with serial imaging and laxatives	No foreign body identified at endoscopy; no mucosal lesion; spontaneous passage
Day 14	2 × AA batteries	Impulsive self-harm; denied primary suicidal intent	GI tract; previous two batteries still in transit	Conservative management + laxatives	Spontaneous passage
Day 30	2 × AA batteries	Patient-described retaliatory behaviour following interpersonal conflict; denied primary suicidal intent	Stomach; persisted on follow-up imaging	Prokinetics followed by upper gastrointestinal endoscopy with endoscopic removal	One battery removed endoscopically; erosive gastropathy identified; remaining battery subsequently passed
Day 38	2 × AA batteries	Patient-described retaliatory behaviour following interpersonal conflict with care team; denied primary suicidal intent	Small bowel/ileum → caecum	Conservative management + laxatives	Spontaneous passage
Day 43	1 × AA battery, re-ingested after passage	Recurrent intentional ingestion; impulsive self-harm; denied primary suicidal intent	Stomach → distal gastrointestinal tract	Conservative management + laxatives + prokinetics	Spontaneous passage
Day 48	Metal screw	Frustration and patient-described retaliatory intent; denied primary suicidal intent	Stomach	Urgent upper gastrointestinal endoscopy	Removed without complication

Throughout these episodes, the patient remained clinically stable, with only mild and intermittent abdominal symptoms documented. Management was coordinated between psychiatry, gastroenterology, and general surgery. Escalation to upper gastrointestinal endoscopy was based on established medical indications and guidelines, including a foreign body requiring retrieval because of its characteristics or anatomical location and persistence of a battery in the stomach on follow-up imaging. In contrast, batteries that had progressed beyond the stomach in a clinically stable patient without evidence of obstruction, perforation, significant gastrointestinal bleeding, or battery casing disruption were managed conservatively with serial clinical and radiological monitoring [4]. Laxatives and prokinetic agents were used when considered appropriate. Battery progression was monitored by serial abdominal radiographs, with passage confirmed by documented direct visualisation of expelled batteries by nursing staff and, when clinically indicated, subsequent imaging. Distally progressing batteries were generally passed spontaneously within approximately five to 10 days. Serial imaging consistently demonstrated intact battery casings, with no documented evidence of disruption or leakage. During the day 31 endoscopy, one gastric battery was removed, and erosive gastropathy was identified; subsequent histology showed chronic non-atrophic gastritis with changes suggestive of chemical gastropathy. Gastroprotective treatment with pantoprazole was maintained during admission. The metal screw was removed endoscopically on day 48. No perforation, intestinal obstruction, significant gastrointestinal bleeding, or other major medical complication occurred.

Following the first two ingestion episodes, four cylindrical batteries were simultaneously present within the gastrointestinal tract, as the batteries from the first episode had not yet been passed when the second ingestion occurred (Figure 1).

**Figure 1:**
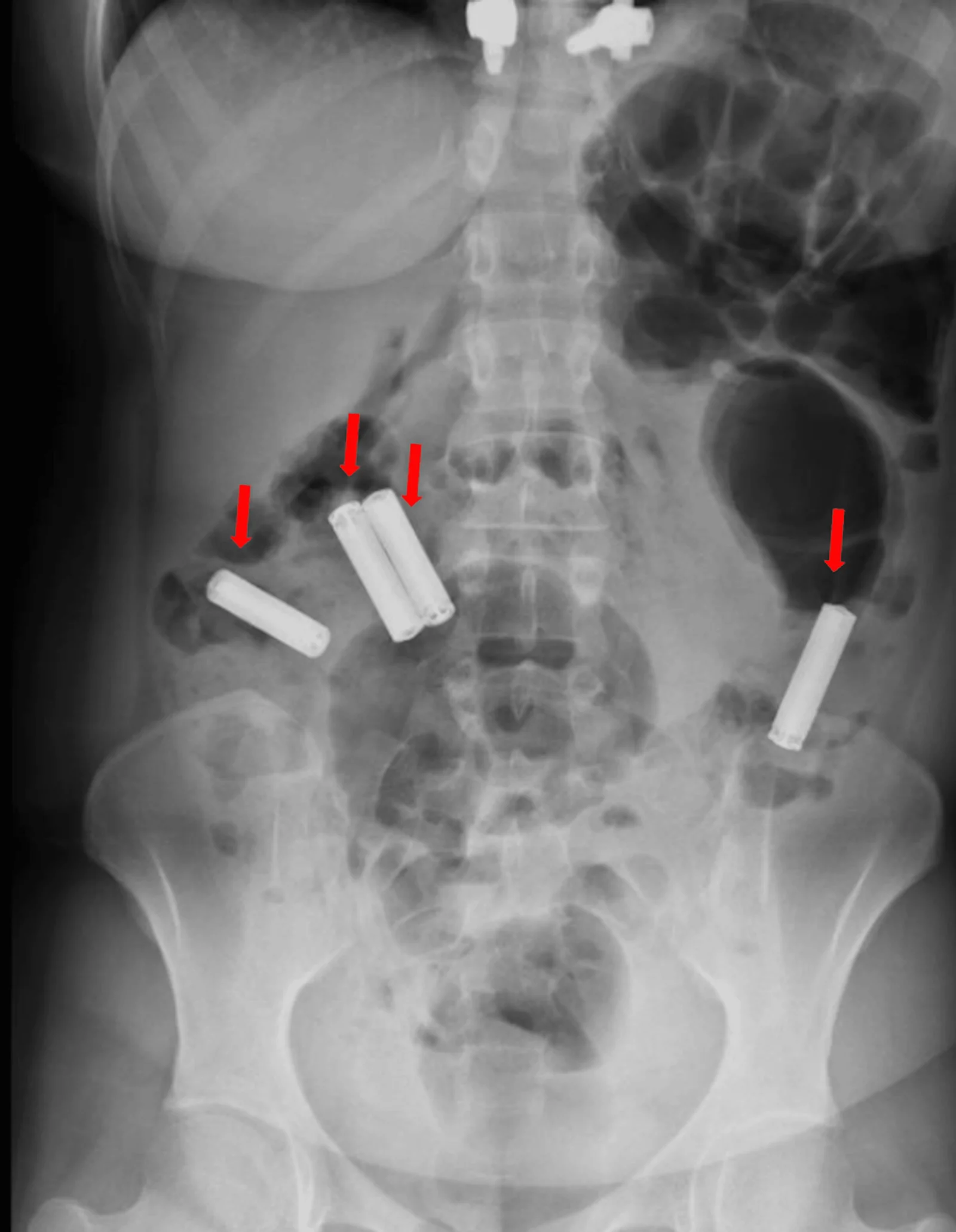
Abdominal radiograph showing ingested cylindrical batteries Abdominal radiograph demonstrating four cylindrical batteries within the gastrointestinal tract, highlighted by red arrows. The radiograph was obtained following the second ingestion episode on Day 14, before the two batteries ingested during the first episode on Day 9 had been completely passed.

The behavioural context of the ingestion episodes evolved during admission. Earlier episodes were characterised as impulsive and reactive self-harm, with limited initial ability to identify precipitating stressors. During later episodes, the patient described some ingestions as retaliatory following perceived interpersonal rejection, particularly in interactions with members of the nursing team when requests for immediate conversation, attention, or engagement in an activity could not be accommodated because of competing clinical duties. The term “retaliatory” reflects the patient's own account of his motivation rather than an independent attribution by the treating team. Assessment of suicidal intent and behavioural function was based on repeated psychiatric evaluations and multidisciplinary observations, integrating the patient's reported intent with the immediate antecedents, affective state, behavioural context, and longitudinal pattern of self-harm, rather than relying solely on denial of suicidal ideation or intent.

Psychiatric assessment throughout admission identified recurrent self-injury, affective dysregulation, interpersonal hypersensitivity, low frustration tolerance, and difficulties identifying and mentalising emotional and interpersonal triggers, in the context of BPD and previously documented factitious disorder. No psychotic symptoms were identified. Possible neurodevelopmental vulnerability was also considered because of sensory sensitivities, motor awkwardness, rocking behaviours, circumscribed interests, rigidity, and repetitive self-injury. The Autism-Spectrum Quotient (AQ) score was 29/50 [8]; however, no formal diagnosis of autism spectrum disorder was established.

During admission, the psychopharmacological regimen was reviewed and adjusted. Topiramate was reduced to 100 mg/day because of concerns regarding possible cognitive adverse effects, flurazepam was progressively reduced and discontinued, and venlafaxine was discontinued. Management otherwise focused on containment of acute risk, identification of precipitating factors, and development of more adaptive self-regulation. Although no structured psychotherapy programme was undertaken during the inpatient admission, several clinical interviews and therapeutic sessions were conducted with family members present. Care was also coordinated with the psychiatrist from the residential psychiatric facility to which the patient was due to return, supporting continuity of psychiatric management after discharge. Over the course of admission, the frequency of self-injurious behaviours decreased, alongside improved self-regulation, greater ability to identify precipitating factors, and increased engagement with proposed interventions.

## Discussion

Deliberate foreign body ingestion is a well-recognised clinical problem among patients with psychiatric disorders, particularly those with personality pathology and recurrent self-injurious behaviour [1,2,3,9]. Repetition is common and may result in substantial use of emergency, endoscopic, surgical, and psychiatric resources [1,3]. Although each ingestion requires an independent medical assessment, recurrent presentations also require attention to the psychological and interpersonal context in which the behaviour occurs.

The medical management of cylindrical battery ingestion differs from that of button battery ingestion. Cylindrical batteries are generally less likely to become lodged and have a different acute injury profile, but prolonged gastric retention, casing damage, leakage, obstruction, or symptoms may necessitate intervention [4-6,10]. Once an intact cylindrical battery has progressed beyond the stomach in a clinically stable patient, conservative management with clinical and radiological follow-up may be appropriate [4-6,10]. Conversely, endoscopic retrieval should not be delayed when established clinical indications are present. Recent case literature further illustrates that management must remain individualised according to battery characteristics, location, symptoms, and progression [11].

In the present case, this medical framework allowed different episodes to be managed differently without compromising safety. Endoscopic retrieval was undertaken when clinically indicated, including when a battery persisted in the stomach on follow-up imaging and for urgent removal of the metal screw, whereas batteries that had progressed distally were managed conservatively with serial imaging and supportive measures. Serial radiological assessments documented intact battery casings without evidence of disruption or leakage. No major medical complications occurred, although erosive gastropathy was identified during one endoscopy, with subsequent histology showing chronic non-atrophic gastritis with changes suggestive of chemical gastropathy. Importantly, the absence of major complications in this patient should not be interpreted as evidence that cylindrical battery ingestion is benign or that conservative management is universally appropriate.

Recurrent intentional foreign body ingestion in patients with personality disorders may serve heterogeneous functions, including self-injury, affect regulation, communication of distress, and responses to interpersonal conflict [1,2,9,12]. In this case, the behavioural context appeared to evolve during admission. Earlier episodes were characterised as impulsive and reactive self-harm, whereas some later episodes were described by the patient as retaliatory in the context of perceived interpersonal rejection. This formulation was based on repeated psychiatric assessments and contemporaneous multidisciplinary observations, integrating the patient's reported intent with immediate antecedents, affective state, interpersonal context, and longitudinal pattern of self-harm. The term “retaliatory” reflects the patient's own account of his motivation rather than an independent attribution by the treating team. This longitudinal variation was clinically relevant because an identical act of ingestion did not necessarily have an identical psychological meaning on each occasion.

This distinction also raises the possibility that aspects of the healthcare response may become incorporated into the behavioural sequence surrounding recurrent ingestion. Repeated emergency assessment, transfer, intensive observation, endoscopy, or other procedures can constitute powerful environmental consequences, although establishing reinforcement in an individual patient is inherently difficult [1,2,12]. Behavioural principles used in the treatment of BPD support attention to antecedents and consequences of recurrent self-harm while avoiding responses that inadvertently strengthen maladaptive behaviours [13]. This does not imply that medically indicated procedures should be withheld. Rather, when more than one medically acceptable option exists, awareness of the behavioural context may help clinicians avoid unnecessary escalation while maintaining appropriate medical surveillance.

Possible neurodevelopmental vulnerability was also considered during admission, with an Autism-Spectrum Quotient (AQ) score of 29/50 [8]. However, the AQ is a screening instrument rather than a diagnostic assessment, and no formal diagnosis of autism spectrum disorder was established. These observations should therefore be interpreted cautiously and should not be used retrospectively to attribute the ingestion behaviour to autism.

The case also highlights the importance of coordination between psychiatry, gastroenterology, surgery, nursing, family members, and the mental health services responsible for subsequent care. Although no structured family psychotherapy programme was undertaken during the inpatient admission, family members participated in several clinical interviews and therapeutic sessions. Care was also coordinated with the psychiatrist from the residential psychiatric facility to which the patient was due to return, supporting continuity of psychiatric management after discharge. A shared management plan can help ensure that each ingestion receives appropriate medical assessment while promoting consistency in communication, observation, and behavioural responses. Psychiatric formulation should complement rather than supersede object-specific and symptom-based medical decision-making.

Several limitations should be acknowledged. This is a single case and cannot establish that conservative management reduces recurrence or that invasive procedures reinforce intentional foreign body ingestion. The behavioural formulation was based on repeated clinical assessments and multidisciplinary observations rather than a standardised functional behavioural assessment and therefore remains interpretative. Similarly, although the patient's account and the temporal relationship between interpersonal events and some later ingestion episodes supported the clinical formulation of retaliatory behaviour, causality cannot be established. Possible neurodevelopmental vulnerability was not confirmed by a formal diagnostic assessment. Furthermore, the favourable outcomes observed with conservative management cannot be generalised beyond patients who meet established criteria for observation. The principal value of this case is therefore not to propose a new medical algorithm, but to illustrate how established medical guidance and longitudinal psychiatric formulation can be integrated in recurrent intentional ingestion.

## Conclusions

Recurrent intentional foreign body ingestion presents a complex challenge at the interface of medical and psychiatric care. Each episode requires assessment according to the characteristics and location of the ingested object, the patient's clinical condition, and established indications for endoscopic or surgical intervention. Psychiatric formulation can add important longitudinal information about the context and possible function of recurrent ingestion, particularly when the behavioural context appears to evolve over time.

Concerns about behavioural reinforcement should never justify withholding a medically necessary procedure. However, when a safe conservative approach is medically appropriate, avoiding unnecessary intervention may form part of a consistent multidisciplinary strategy. This case illustrates an integrated approach in which medical safety remains the primary determinant of management, while psychiatric and behavioural understanding informs proportionate, coordinated care.
